# Abetalipoproteinemia Due to a Novel Splicing Variant in *MTTP* in 3 Siblings

**DOI:** 10.1177/23247096211022484

**Published:** 2021-06-02

**Authors:** Caitlyn Vlasschaert, Adam D. McIntyre, Lauren A. Thomson, Brooke A. Kennedy, Suzanne Ratko, Chitra Prasad, Robert A. Hegele

**Affiliations:** 1 Queen’s University, Kingston, Ontario, Canada; 2 Western University, London, Ontario, Canada; 3 Children’s Hospital—London Health Sciences Centre, London, Ontario, Canada

**Keywords:** apolipoprotein B, neuropathy, fat-soluble vitamin deficiency, malabsorption

## Abstract

Abetalipoproteinemia (ABL) is a rare recessive condition caused by biallelic loss-of-function mutations in the *MTTP* gene encoding the microsomal triglyceride transfer protein large subunit. ABL is characterized by absence of apolipoprotein B–containing lipoproteins and deficiencies in fat-soluble vitamins leading to multisystem involvement of which neurological complications are the most serious. We present 3 siblings with ABL who were born to non-consanguineous parents of Filipino and Chinese background. Identical twin boys with long-standing failure to thrive and malabsorption were diagnosed at age 2 years. ABL therapy with vitamins and a specialized diet was initiated, replacing total parenteral nutrition at age 3 years. Their younger sister was diagnosed from a blood sample taken at birth; treatment was instituted shortly thereafter. We observed in the twins reversal and in their sister prevention of ABL systemic features following early implementation of fat restriction and high doses of oral fat-soluble vitamins. A targeted sequencing panel found that each affected sibling is homozygous for a novel *MTTP* intron 13 -2A>G splice acceptor site mutation, predicted to abolish splicing of intron 13. This variant brings to more than 60 the number of reported pathogenic mutations, which are summarized in this article. The twin boys and their sister are now doing well at 11 and 4 years of age, respectively. This experience underscores the importance of early initiation of targeted specialized dietary and fat-soluble vitamin replacements in ABL.

## Introduction

**Figure 1.**
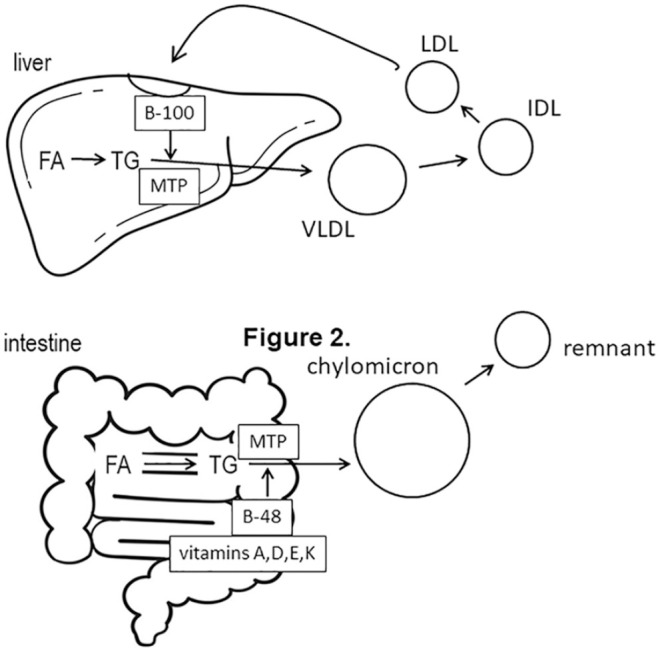
Microsomal triglyceride (TG) transfer protein (MTP) and lipoprotein assembly. Fatty acids (FA) in the intestine or liver are assembled into TG by diacylglycerol acyltransferases. In enterocytes, MTP combines TG, cholesterol, phospholipids, dietary fat–soluble vitamins with the B-48 intestine-specific isoform of apolipoprotein (apo) B to form chylomicrons that enter the plasma through intestinal lymphatics and are catabolized to remnant particles by lipoprotein lipase. MTP activity in hepatocytes is similar except the liver-specific B-100 isoform is incorporated into very-low-density lipoprotein (VLDL) particles, which are catabolized to intermediate-density lipoprotein (IDL) and ultimately low-density lipoprotein (LDL) particles by plasma lipases. LDL is removed from plasma by hepatic LDL receptors. Biallelic mutations that abolish MTP activity in abetalipoproteinemia result in complete absence of the above apo B–containing lipoproteins. The associated deficiency of fat-soluble vitamins occurs because chylomicrons are unavailable to transport them.

Abetalipoproteinemia (ABL; OMIM 200100) is an autosomal recessive disorder whose core biochemical defect is impaired absorption of dietary lipids with impaired assembly and secretion of apolipoprotein (apo) B–containing lipoprotein particles.^1^ Biallelic pathogenic mutations have been reported in all functional domains of the *MTTP* gene that encodes the microsomal triglyceride (TG) transfer protein large subunit (MTP) in ABL patients.^2^ MTP plays a critical role in the assembly of lipoproteins. In the intestine and liver MTP transfers lipid to apo B-48 and apo B-100, respectively, to form chylomicrons and very-low-density lipoprotein (VLDL), respectively (Figure 1).^3^ The impaired VLDL secretion results in severe reduction of its remodeled end product in plasma, namely, low-density lipoprotein (LDL).^4^ The absence of functional MTP in ABL manifests as extremely low to undetectable levels of apo B–containing lipoproteins, total and LDL cholesterol, TG, and of plasma biomarkers reflecting the status of vitamins A, D, E, and K.^5^

ABL patients typically present in early childhood with failure to thrive and steatorrhea.^1^ Chronic vitamin deficiencies produce serious physiologic and developmental consequences, including retinal degeneration from vitamin A deficiency, osteomalacia and osteoporosis from vitamin D deficiency, and prolonged international normalized ratio from vitamin K deficiency.^1^ Chronic vitamin E deficiency results in severe, progressive neuromuscular deficits.^1^ Individuals with untreated ABL typically develop muscle weakness, dysarthria, and a loss of deep tendon reflexes, vibratory sense, and proprioception in childhood, and which later progresses to Friedreich’s-like spinocerebellar ataxia.^1,5,6^ Mild hemolytic anemia develops secondary to acanthocytosis. The differential diagnosis of ABL includes homozygous hypobetalipoproteinemia^1,5^ and chylomicron retention disease^7^ due to biallelic rare mutations in the *APOB* and *SAR1B* genes, respectively. These conditions can be differentiated from ABL using both biochemical analyses and diagnostic DNA sequencing.^8^

Treatment of ABL involves the following: (1) dietary restriction of fats to improve malabsorption symptoms and (2) supplementation with high-dose oral fat-soluble vitamins to avert complications.^5^ Delayed diagnosis and treatment of ABL results in delayed growth and development along with irreversible ophthalmologic and neurologic complications.^5,6^ We present a unique Filipino/Chinese Canadian family with 3 siblings—identical twin boys and their sister—who were born to non-consanguineous parents and were diagnosed with ABL. Each was homozygous for a previously unreported rare *MTTP* intron 13 -2A>G variant, which was predicted to abolish normal RNA splicing. Treatment for the boys was started at age 3 years and for their younger sister was started shortly after birth, with successful long-term outcomes to date.

## Subjects and Methods

### Probands

**Figure 2.**
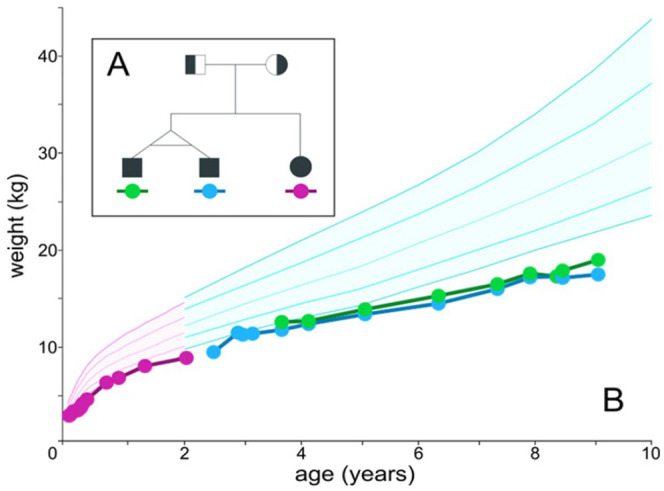
(A) Pedigree of abetalipoproteinemia (ABL) family. The parents are each carriers of one copy of the same mutant allele (*MTTP* intron 13 -2A>G). Their children, identical twin boys, and their younger sister are homozygous for this mutant allele. (B) Growth trajectories of the 3 siblings affected by ABL. Siblings are identified as indicated and are described clinically in the text.

Identical 3-year-old twin boys of East and South East Asian ancestry were referred to our lipid clinic with a history of failure to thrive and steatorrhea from shortly after birth (Figure 2A). A clinical diagnosis of ABL was made at age 18 months at another center and total parenteral nutrition (TPN) was subsequently initiated. There were no specific restrictions on oral intake. At the time of presentation to our clinic, the twins were severely underweight with reduced appetite (Figure 2B), low energy, easy fatigability, and significant muscle weakness along with developmental delay for most milestones. There were no visual symptoms, nor was there a history of easy bruising or bleeding. In addition, both twins had persistent abdominal bloating and daily large volume steatorrhea. For both twins, serum levels of vitamins A and E were undetectable, vitamin D was at the lower limit of normal, while international normalized ratio was normal in both at 1.1.

After assessment in our clinic, the parents were advised to discontinue TPN and initiate a more varied diet, but with restriction of fat to 20% of total calories. High doses of oral vitamins were also initiated: vitamin A 20 000 IU daily, vitamin D 10 000 IU 5 days per week, vitamin E 2400 U daily, and vitamin K 5 mg per week. Within 2 months, the parents reported that both boys were more alert, mobile, and energetic, with reduced abdominal distension and less frequent bowel movements. Within a year, they attended preschool and thereafter primary school at a grade level appropriate for their age, with full participation in academic and nonacademic activities. When the twins were 9 years old, a fatty acid supplement was added, that is, eicosapentanoic acid and docosahexanoic acid in a 1:2 ratio, for a total of 50 mg docosahexanoic acid daily.

The twin boys have remained continuously on this regimen of high-dose oral fat-soluble vitamins with dietary fat restriction for 8 years. While they remain underweight (less than third percentile; Figure 2B), they have regained functional strength and dexterity, and have caught up to their peers’ developmental milestones. Their fat-soluble vitamin plasma levels—most importantly vitamins A and E—are now each within the detectable range, although vitamin E levels for both are low but stable at 3 µmol/L (reference range 18-29 µmol/L). Plasma vitamin levels are monitored every 6 months and supplementation doses are adjusted as required to maintain appropriate levels.^5^

A female full sibling was born when the twin boys were 7 years old (Figure 2A). She was diagnosed with ABL via molecular analysis of cord blood at 3 weeks of age. She was exclusively breast fed for the first month of life, and she gradually transitioned to a fat-restricted diet with high-dose medium-chain fatty acids (MCFA) and fat-soluble vitamin supplementation by 4 months. She is developmentally normal and has grown at or above the third percentile for weight. Currently, she takes vitamin A 10 000 IU daily, vitamin D 1000 IU 5 days weekly, vitamin E 1600 IU daily, and vitamin K 2.5 mg weekly. Her most recent plasma levels of vitamins A and D are normal, while vitamin E was 3 µmol/L (reference range 18-29 µmol/L). All 3 children undergo yearly neurological and ophthalmologic examinations as well as abdominal ultrasound examinations to screen for hepatosteatosis; all medical monitoring has been normal to date.

### DNA Sequencing and Genetic Analysis

Each sibling provided a venous blood sample from which genomic DNA was extracted. We used targeted next-generation pandyslipidemia sequencing panel called LipidSeq, as described.^9-13^ Genotype status of the probands and parents was confirmed by Sanger sequencing, as described.^9-13^

## Results

### Genetic Analysis

**Figure 3.**
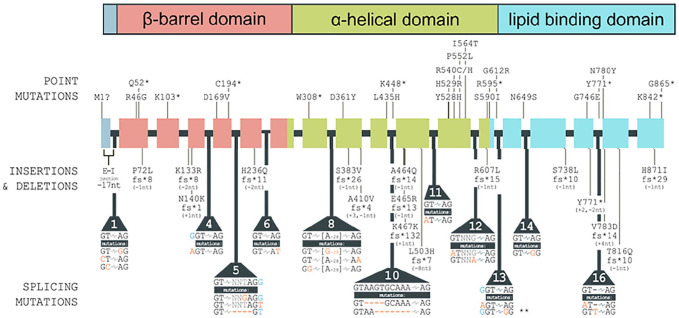
Synopsis of reported *MTTP* mutations causing abetalipoproteinemia. The top panel shows the functional domains of the MTP protein, which are encoded by the indicated regions of the *MTTP* gene depicted with its 18 exons and 17 introns in the middle of the figure. Point mutations, frame-shifting insertions and deletions, and splicing mutations are indicated and occur throughout the *MTTP* gene, affecting all functional domains of the MTP protein. Large-scale deletions (≥25 base pairs) and *MTTP* mutations linked to other conditions are not included in the figure.

Targeted next-generation sequencing revealed in all 3 siblings a novel homozygous splicing acceptor variant that was predicted to be deleterious; namely, *MTTP* c.1990 -2A>G. Both asymptomatic parents were heterozygous for this variant. This mutation was absent from the ExAC, 1000G, and ESP databases.^14,15^ The reported *MTTP* mutations in ABL patients are summarized in Figure 3.

## Discussion

We present a family of East and South East Asian background with the unusual co-occurrence (1 in 64 probability) of all 3 siblings affected with ABL. Despite no known relationship between the parents, each child was homozygous for the identical rare unreported *MTTP* c.1990 -2A>G pathogenic variant, which is predicted to have compromised function in RNA splicing occurring within a splice acceptor sequence in intron 13. The clinical course of these children indicates that high-dose oral vitamin supplementation and fat restriction can reverse the disease trajectory if initiated at 3 years of age, and can prevent systemic abnormalities when initiated shortly after birth.

Switching the twin boys from TPN to oral diet with high-dose fat-soluble vitamins resulted in generalized clinical improvement. While TPN formulations contain lipids and fat-soluble vitamins, the quantities are insufficient and physiologically inappropriate to affect the severe deficiencies that are characteristic of ABL. Current treatment guidelines concur with many years of clinical experience and recommend high oral doses of fat-soluble vitamin supplementation as a foundation of treatment.^1,5^ The extent of total intestinal fat absorption is low in ABL due to the profound defect in chylomicron synthesis. However, MCFAs can be directly absorbed into the hepatic portal vein system, independent of chylomicron transport^1,5,6^ and can provide a source of calories if clinically indicated. Furthermore, a small proportion of high doses of orally administered vitamins A, D, E, and K can enter the portal vein through the MCFA pathway.^1,5,6^

The twin boys exhibited growth and developmental delays prior to initiation of standard treatment, but these have reversed with treatment. Now at age 11 years, their eating habits and bowel routine has essentially normalized. Both are doing well in school and extracurricular activities, although they still lag behind normal trajectories for height and weight. Their younger sister, now age 4 years, was diagnosed days after birth and was started on fat restriction with MCFA supplementation in the first 2 months of life, with high-dose fat-soluble vitamins initiated shortly thereafter. She maintains appropriate global development and healthy dietary behaviors to date. The findings here indicate that early initiation of proper life-sustaining therapy in ABL allows for ongoing appropriate growth and development.
